# Antibacterial Effect of Functionalized Polymeric Nanoparticles on Titanium Surfaces Using an In Vitro Subgingival Biofilm Model

**DOI:** 10.3390/polym14030358

**Published:** 2022-01-18

**Authors:** Jaime Bueno, Leire Virto, Manuel Toledano-Osorio, Elena Figuero, Manuel Toledano, Antonio L. Medina-Castillo, Raquel Osorio, Mariano Sanz, David Herrera

**Affiliations:** 1 ETEP (Etiology and Therapy of Periodontal and Peri-Implant Diseases) Research Group, University Complutense, Pza. Ramón y Cajal s/n, 28040 Madrid, Spain; jaimebue@ucm.es (J.B.); lvirto@ucm.es (L.V.); elfiguer@ucm.es (E.F.); marsan@ucm.es (M.S.); davidher@ucm.es (D.H.); 2 Faculty of Dentistry, University of Granada, Colegio Máximo de Cartuja s/n, 18071 Granada, Spain; mtoledano@correo.ugr.es (M.T.-O.); toledano@ugr.es (M.T.); 3 Faculty of Sciences, University of Granada, Colegio Máximo de Cartuja s/n, 18071 Granada, Spain; amedina@nanomyp.com

**Keywords:** polymers, nanoparticles, doxycycline, antibacterial, zinc, calcium, biofilm

## Abstract

This investigation aimed to evaluate the antibacterial effect of polymeric nanoparticles (NPs), functionalized with calcium, zinc, or doxycycline, using a subgingival biofilm model of six bacterial species (*Streptococcus oralis,* *Actinomyces naeslundii, Veillonela parvula, Fusobacterium nucleatum, Porphyromonas gingivalis,* and *Aggregatibacter actinomycetemcomitans*) on sandblasted, large grit, acid-etched titanium discs (TiDs). Undoped NPs (Un-NPs) or doped NPs with calcium (Ca-NPs), zinc (Zn-NPs), or doxycycline (Dox-NPs) were applied onto the TiD surfaces. Uncovered TiDs were used as negative controls. Discs were incubated under anaerobic conditions for 12, 24, 48, and 72 h. The obtained biofilm structure was studied by scanning electron microscopy (SEM) and its vitality and thickness by confocal laser scanning microscopy (CLSM). Quantitative polymerase chain reaction of samples was used to evaluate the bacterial load. Data were evaluated by analysis of variance (*p* < 0.05) and post hoc comparisons with Bonferroni adjustments (*p* < 0.01). As compared with uncovered TiDs, Dox-NPs induced higher biofilm mortality (47.21% and 85.87%, respectively) and reduced the bacterial load of the tested species, after 72 h. With SEM, scarce biofilm formation was observed in Dox-NPs TiDs. In summary, Dox-NPs on TiD reduced biofilm vitality, bacterial load, and altered biofilm formation dynamics.

## 1. Introduction

Different strategies have been developed over the years to rehabilitate lost dentition. The current use of dental implants has demonstrated long-term survival and success [1], but also frequent complications [2], as peri-implant diseases, which are chronic inflammatory conditions of the peri-implant tissues. When the inflammatory lesion affects the peri-implant mucosa, without loss of supporting bone, the condition is diagnosed as peri-implant mucositis [3], whereas, when the inflammation also results in progressive loss of supporting bone, the diagnosis is peri-implantitis [4], and can eventually lead to the loss of the implant [5].

The prevalence of peri-implant diseases is high, ranging between 19% and 65% in the case of peri-implant mucositis, and between 1% and 47% for peri-implantitis [6,7,8], what makes prevention, early diagnosis, and treatment of these conditions very important.

Although the etiology of peri-implant diseases is multifactorial, the accumulation of bacterial biofilms on the implant crown, abutment, and implant surfaces is the most important factor [5]. A history of periodontitis has been shown to be a risk factor for peri-implantitis, and this inflammatory condition is usually more aggressive [9,10] and its treatment less predictable and efficacious as compared with the treatment of periodontitis [11,12]. Thus, distinct strategies have been proposed for the treatment of peri-implantitis, mainly through the decontamination of biofilm contaminated surfaces by means of debridement/instrumentation of these surfaces, with or without adjunctive systemic or local antimicrobial agents [13]. However, the predictability of these treatment protocols remains to be a challenge, and future research is seeking new approaches and adjunctive agents.

In recent years, nanotechnology has gained relevance in medicine and dentistry for its use in prevention, diagnosis, and treatment of different conditions [14,15,16]. Different nanostructured materials have been proposed for the treatment of periodontal and peri-implant diseases [17,18], such as polymeric nanoparticles due to their antimicrobial activity [19,20]. These polymeric nanoparticles (NPs) are non-resorbable and exhibit carboxyl groups on their external surface, which may be functionalized with different molecules, thus, enhancing their antibacterial properties. For example, they can effectively chelate and release calcium and zinc [19,21]. Similarly, doxycycline loaded NPs have been shown to release most of this antibacterial agent (around 70%) up to 28 d [21]. These functionalized NPs act as antibacterial agents not only on planktonic cultures, but also in subgingival biofilms when grown on hydroxyapatite discs [20,21], which makes their use a potentially effective tool adjunctive to the mechanical treatment of peri-implant diseases.

The objective of this in vitro investigation was to evaluate the antibacterial capacity of NPs functionalized with zinc, calcium, and doxycycline, in a validated in vitro oral biofilm model over titanium discs with sandblasted, large grit, and acid-etched (SLA) surface. As specific objectives, the bacterial load, the vitality and thickness of the biofilms were analyzed.

## 2. Materials and Methods

### 2.1. Bacterial Strains and Culture Conditions

The bacterial strains *Streptococcus oralis* CECT 907T, *Veillonella parvula* NCTC 11810, *Actinomyces naeslundii* ATCC 19039, *Fusobacterium nucleatum* DMSZ 20482, *Aggregatibacter actinomycetemcomitans* DSMZ 8324, and *Porphyromonas gingivalis* ATCC 33277 were selected for bacterial growth in blood agar plates (Blood Agar Oxoid N° 2, Oxoid, Basingstoke, UK), supplemented with 5% (*v*/*v*) sterile horse blood (Oxoid), 5.0 mg L-1 hemin (Sigma, St. Louis, MO, USA), and 1.0 mg L-1 menadione (Merck, Darmstadt, Germany), under anaerobic conditions (10% H_2_, 10% CO_2_, and balance N_2_) at 37 °C for 24–72 h.

### 2.2. Nanoparticle Production

Four different NPs, developed as previously described by Osorio et al. [19], were studied: (a) Undoped NPs (Un-NPs), (b) NPs loaded with zinc (Zn-NPs), (c) NPs loaded with calcium (Ca-NPs), and (d) NPs doped with doxycycline (Dox-NPs). NPs were created through a polymerization/precipitation procedure with a composition of 2-hydroxyethyl methacrylate (backbone monomer), ethylene glycol dimethacrylate (cross-linker), and methacrylic acid (functional monomer), with a final diameter of approximately 150 nm [22,23]. For the functionalizing process, 30 mg of Zn-NPs and Ca-NPs were immersed for 3 days at room temperature, and under continuous agitation, in an aqueous solution of ZnCl_2_ and CaCl_2_ (containing zinc and calcium at 40 ppm, at pH 6.5) until reaching the adsorption equilibrium of metal ions. Next, the particles were removed from the supernatant and suspended in phosphate buffered saline (PBS). Subsequently, the suspensions were centrifuged for 20 min (6000× *g*), and the particles were detached from the supernatant. The same centrifugation technique, with the addition of PBS was used to wash the samples, and it was repeated twice. Ion complexation values were 2.15 ± 0.05 μg Zn/mg NPs and 0.96 ± 0.04 μg Ca/mg NPs, respectively [21]. For doping NPs with doxycycline, 30 mg of nanoparticles were submerged in a 40 mgL^−1^ aqueous solution of doxycycline hyclate (Sigma-Aldrich, Chemie Gmbh, Riedstr, Germany). NPs were maintained for 30 min under constant shaking. The achieved amount of doxycycline per gram of NPs was 70 μg [21]. As with the other NPs, the suspensions were centrifuged. The NPs were separated from the supernatant and re-suspended in PBS [21]. Doped NPs have been previously shown to effectively liberate zinc, calcium, and doxycycline [21].

### 2.3. Specimen Production

Sterile titanium discs (TiDs) (grade 2) of 5 mm of diameter (manufactured and donated by Straumann, Institut Straumann AG, Basel, Switzerland) with surfaces comparable to the commercially available SLActive^®^ surface (Institut Straumann AG) were used. The different types of NPs were all diluted in PBS (10 mg/mL) and were applied onto the surfaces of the TiDs. Discs coated with PBS without NPs were used as the control.

### 2.4. Biofilm Development on the Prepared Specimens

The surfaces of TiDs covered with Un-NPs, Zn-NPs, Ca-NPs, Dox-NPs, and PBS were used for the establishment of the multispecies biofilms [24]. Pure cultures of each bacterium were grown anaerobically in a medium containing a high concentration of proteins formed by brain heart infusion (BHI) (Becton, Dickinson and Company, Franklin Lakes, NJ, USA) supplemented with 2.5 g L^−1^ mucin (Oxoid), 1.0 g L^−1^ yeast extract (Oxoid), 0.1 g L^−1^ cysteine (Sigma), 2.0 g L^−1^ sodium bicarbonate (Merck), 5.0 mg L^−1^ hemin (Sigma, St. Louis, MO, USA), 1.0 mg L^−1^ menadione (Merck, Darmstadt, Germany) and 0.25% (*v*/*v*) glutamic acid (Sigma). The bacteria were collected at the mid-exponential phase of bacterial growth (measured by spectrophotometry) and a mixed bacterial suspension was prepared in modified BHI medium containing 10^3^ colony forming units (CFU) mL^−1^ of *S. oralis*, 10^5^ CFU mL^−1^ of *V. parvula* and *A. naeslundii*, and 10^6^ CFU mL^−1^ of *F. nucleatum, A. actinomycetemcomitans,* and *P. gingivalis*.

Then, 1.5 mL of mixed bacteria suspension was placed over TiDs coated with PBS or with the different tested products, in a multi-well plate of a 24-well tissue culture plate (Greiner Bio-one, Frickenhausen, Germany) and were incubated in anaerobic conditions (10% H_2_, 10% CO_2_, and balance N_2_) at 37 °C for 12, 24, 48, and 72 h. Plates containing only culture medium were also cultured in order to be sure of the sterility of the culture medium.

### 2.5. Morphological Analysis of Biofilms by Scanning Electron Microscope (SEM)

Biofilms were analyzed by SEM at different times of growth (12, 24, 48, and 72 h). Specimen fixation was performed by immersion in a 4% paraformaldehyde and 2.5% glutaraldehyde solution for 4 h, at 4 °C. Then, the discs were washed with PBS and sterile water (10 min each) and submitted to critical point drying. Specimens were sputter-coated with gold and analyzed by SEM using a JSM 6400 (JSM6400, JEOL, Tokyo, Japan), with a back-scattered electron detector at an image resolution of 25 kV.

### 2.6. Analysis of Biofilms’ Vitality and Thickness by Confocal Laser Scanning Microscopy (CLSM)

The non-invasive confocal imaging of fully hydrated biofilms was carried out by means of a fixed-stage Ix83 Olympus inverted microscope coupled to an Olympus FV1200 confocal system (Olympus, Shinjuku, Tokyo, Japan). The objective lens was a ×63 water-immersion lens (Olympus). Specimens were stained at room temperature with LIVE/DEAD^®^ BacLight^TM^ Bacterial Viability Kit solution (L7012, Molecular Probes B. V., Leiden, The Netherlands). A staining time of 8 ± 1 min, in a 1:1 fluorochrome ratio was used to obtain the best fluorescence signal at the corresponding wavelengths (Syto9, 515–530 nm and propidium iodide, PI > 600 nm). At least three different and demonstrative locations of the discs were selected for the study. A z-series of scans (xyz) of 1 μm thickness (8 bits, 1024 × 1024 pixels) were analyzed thanks to the configuration of the CLSM control software. Image stacks were analyzed by using the Olympus^®^ software (Olympus^®^). To quantify the biomass and cell viability within the biofilm, total fluorescent staining of the confocal micrographs was analyzed using an open source image analysis software (Fiji ImageJ) by measuring voxel intensities from two-channel images and, thus, calculating the percentage of the biomass and cell viability within the stacks [25].

### 2.7. DNA Isolation and Quantitative Polymerase Chain Reaction (qPCR)

DNA of the biofilms at 12, 24, 48, and 72 h was obtained from all samples using a commercial kit (D-321-100, MolYsis Complete5, Molzym GmgH & CoKG, Bremen, Germany), following the manufacturer’s instructions. The qPCR technique with hydrolysis probes was used to detect and quantify the bacterial DNA. The primers and probes were obtained by Life Technologies Invitrogen (Carlsbad, CA, USA), Applied Biosystems (Carlsbad, CA, USA) and Roche (Roche Diagnostic GmbH, Mannheim, Germany) and were fixed against the 16S rRNA gene. A total volume of 10 μL of the reaction mixture was used for the amplification of the qPCR. The reaction mixtures contained 5 μL Master Mix 2x (LC 480 Probes Master, Roche), optimal primers and probe concentrations [25] (900, 900, and 300 nM for *S. oralis*; 300, 300, and 300 nM for *A. naeslundii*; 750 and 400 nM for *V. parvula*; 300, 300, and 200 nM for *A. actinomycetemcomitans*; 300, 300, and 300 nM for *P. gingivalis;* and 600, 600, and 300 nM for *F. nucleatum*), as well as 2 μL of DNA of the corresponding samples. The negative control was 2 μL of sterile water (Water PCR grade, Roche). The samples were subjected to an initial amplification cycle at 95 °C for 10 min, followed by 45 cycles at 95 °C for 15 s and 60 °C for 1 min. The analyses were performed with a LightCycler^®^ 480 II thermal cycler (Roche). Plates LightCycler^®^ 480 Multiwell Plate 384 (Roche), sealed with qPCR Adhesive Clear Seals (4titude), were employed.

Each DNA sample was analyzed in duplicate. The value of the quantification cycle (Cq) was determined using a computer software (LC 480 Software 1.5, Roche Diagnostic GmbH, Mannheim, Germany) based on standard curves. The correlation between the Cq values and the CFU mL^−1^ was generated automatically through the software (LC 480 Software 1.5, Roche).

### 2.8. Data Analysis

The primary outcome variable to compare biofilms formed over TiD surfaces exposed to the different NPs was CFUs mL^−1^ of the six bacterial species presented in the biofilms at 12, 24, 48, and 72 h. The secondary outcomes were cell vitality (72 h), thickness (72 h), and morphological appearance of the biofilms (12, 24, 48, and 72 h).

Data were expressed as means and standard deviations (SD). The Kolmogorov–Smirnov test was used to assess data normality. The non-parametric analyses Kruskal-Wallis ANOVA and pairwise Mann–Whitney comparisons were used. Cell vitality and the thickness of the formed biofilms at 72 h (*n* = 6) on the different treated discs were compared to the control group and also the effects of NPs at different exposure times on CFU mL^−1^ (*n* = 9), in this case, the different time periods were analyzed separately. Statistical significance was set at *p* < 0.05, except for post hoc comparisons, where a Bonferroni correction was applied, and significance was set at *p* < 0.01. The morphological appearance of the biofilms, in the different groups, was described as a secondary outcome variable. The software package (IBM SPSS Statistics 24.0, IBM Corporation, Armonk, NY, USA) was used for all data analysis.

## 3. Results

### 3.1. Morphological Analysis of Biofilms by Scanning Electron Microscope (SEM)

**Figure 1:**
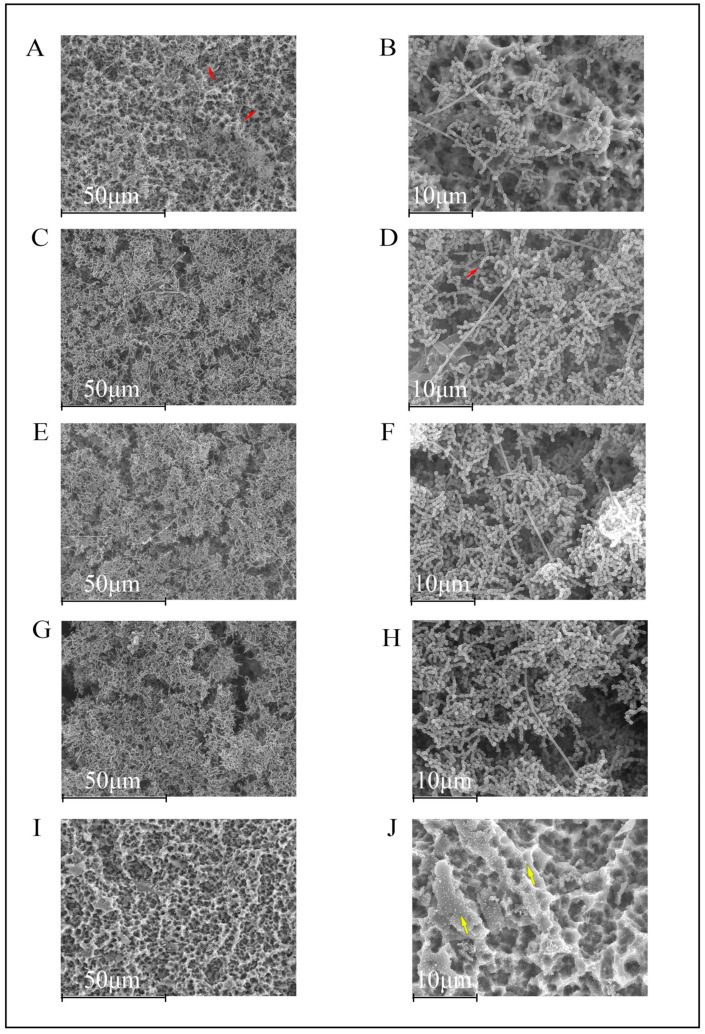
Scanning electron microscopy images of 12 h biofilms on titanium discs (TiDs). *F. nucleatum* is recognized due to its morphological fusiform bacillus appearance. Bacteria colonized the entire rough surface of the phosphate buffer saline (PBS, negative control) coated TiDs (**A**,**B**). TiDs covered with undoped nanoparticles (NPs) (**C**,**D**), calcium nanoparticles (**E**,**F**), and zinc nanoparticles (**G**,**H**) presented a similar pattern of bacterial presence and distribution. The surfaces of TiDs covered with doxycycline nanoparticles (**I**,**J**) did not present biofilm formation. Bacilli and cocci are marked with red arrows. The NPs are sometimes visible (yellow arrows). Magnification: (**A**,**C**,**E**,**G**,**I**) 1000×; (**B**,**D**,**F**,**H**,**J**) 3000×.

After 12 h of biofilm growth, the SLA TiD surfaces in the negative control group showed the typical morphology of early biofilm formation, with the presence of individual bacterial cells, bacterial chains, and bacterial co-aggregates (Figure 1A,B). At this stage, *F. nucleatum* was easily identified through its characteristic fusiform bacillus appearance.

TiDs covered with Un-NPs, Ca-NPs, and Zn-NPs presented a similar pattern of biofilm formation, although with lesser bacterial biomass as compared with the negative control group (uncovered TiDs) (Figure 1C–H). However, as compared with TiDs covered with Dox-NPs, these TiDs lacked biofilm formation, with evidence of the presence of NPs coating the SLA titanium surfaces (Figure 1I,J).

**Figure 2:**
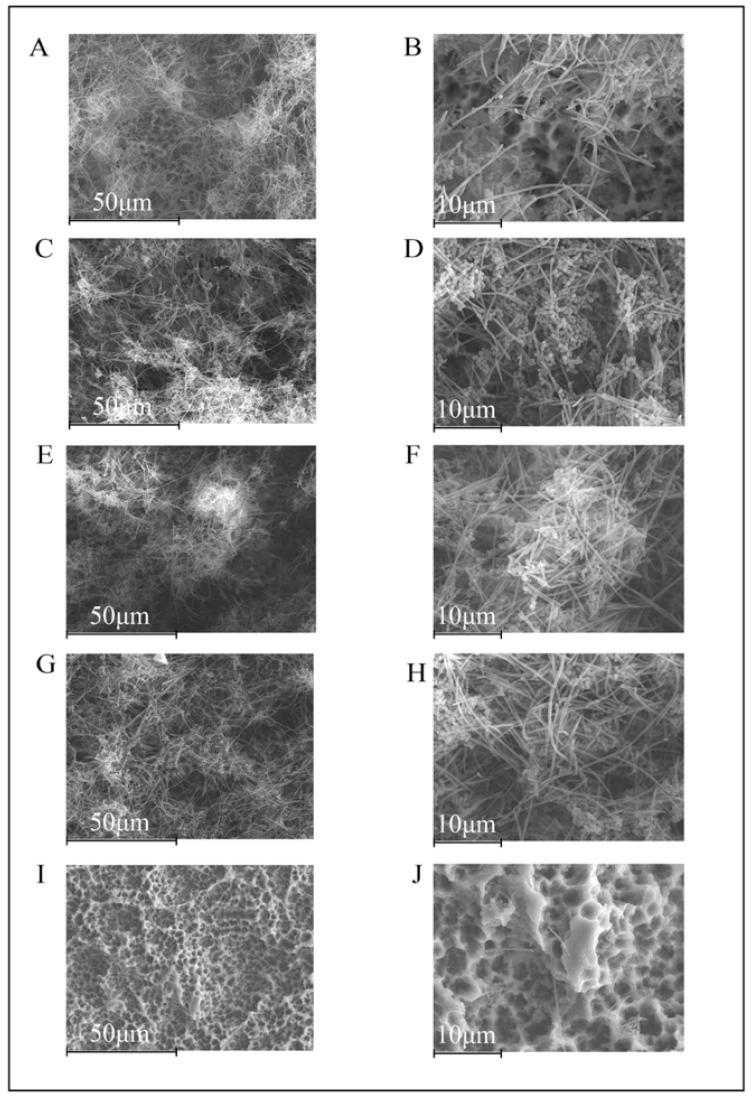
Scanning electron microscopy images of biofilms, after 24 h of development on titanium discs (TiDs). Discs with undoped nanoparticles (**C**,**D**), calcium nanoparticles (**E**,**F**), and zinc nanoparticles (**G**,**H**), demonstrated a similar biofilm structure (**A**,**B**), with higher amounts of bacteria, and covering the entire surface of the TiDs. However, the discs with doxycycline nanoparticles evidenced the surfaces of the TiDs free from biofilm formation, with only a few isolated bacterial cells (**I**,**J**). Magnification: (**A**,**C**,**E**,**G**,**I**) 1000×; (**B**,**D**,**F**,**H**,**J**) 3000×.

As compared with 12-hour biofilms, the 24-hour biofilms in the negative control group presented thicker biomass and larger amounts of *F. nucleatum* (Figure 2A,B). In Un-NPs, Zn-NPs, and Ca-NPs, the structure was similar, but with a higher bacteria load (Figure 2C–H). In the Dox-NPs treated surfaces, there was no biofilm formation, only depicting some isolated bacterial cells on the disc surfaces (Figure 2I–J).

**Figure 3:**
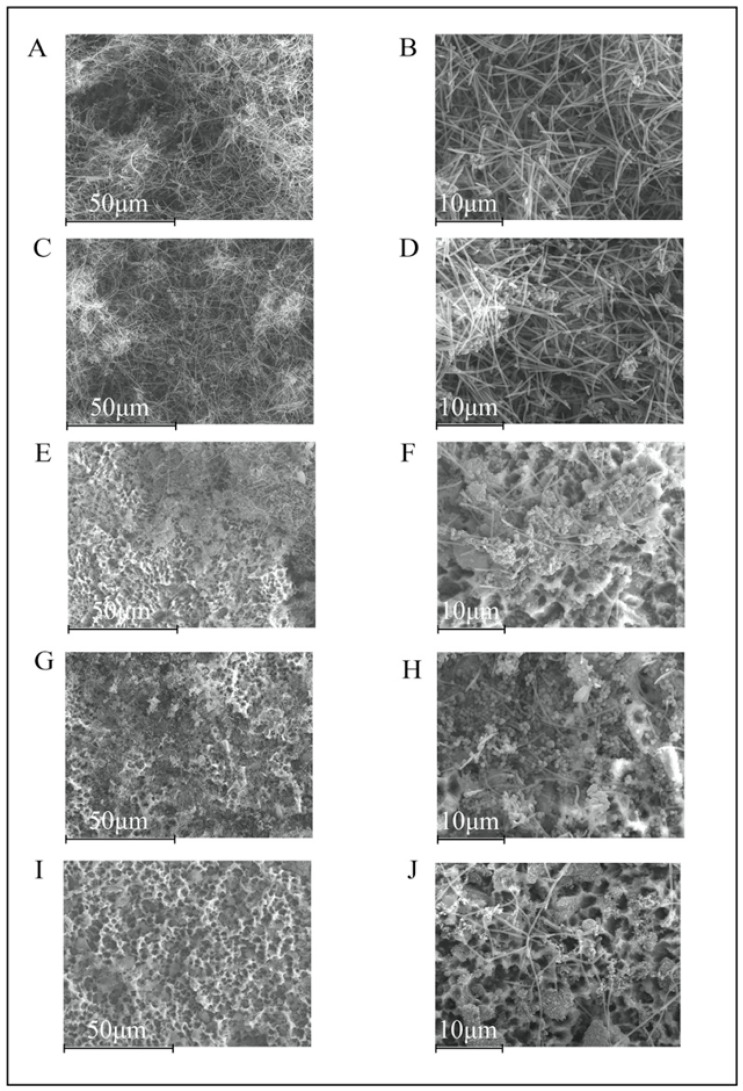
Scanning electron microscopy images of 48 h biofilms depicting increased area of the titanium disc covered by bacteria. In the negative control group (**A**,**B**) and the undoped nanoparticles (NPs) (**C**,**D**), the biofilm was spread throughout the disc surface, while in calcium-doped NPs (**E**,**F**) and zinc-doped NPs (**G**,**H**), some areas of titanium were still uncovered. Titanium disc covered with doxycycline-doped NPs (**I**,**J**) did not show biofilm formation, but rather a few scattered visible cells. Magnification: (**A**,**C**,**E**,**G**,**I**) 1000×; (**B**,**D**,**F**,**H**,**J**) 3000×.

At 48 h, the morphological characteristics of the biofilms in the negative control discs were similar, with evidence of bacterial stacks and tunnel formation, the typical features of mature biofilms (Figure 3A,B). In the Un-NPs group, the entire surface of the TiD was covered with biofilm growth, in contrast with Ca-NPs and Zn-NPs TiDs, where some areas of titanium free of bacterial growth were observed (Figure 3C–H). The TiDs covered with Dox-NPs was again free of biofilm formation, with the presence of only a few scattered deposits and cells were visible (Figure 3I,J).

**Figure 4:**
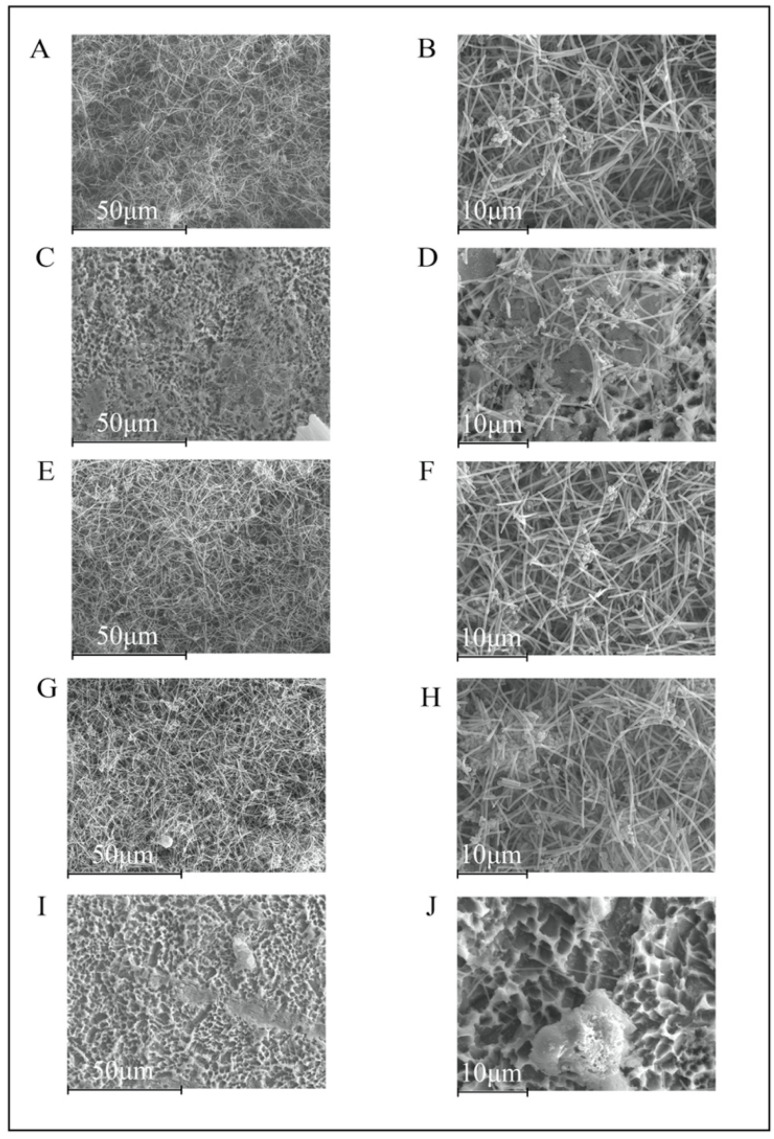
Scanning electron microscopy images of 72 h biofilms on titanium discs coated with: (**A**,**B**) phosphate buffered saline (PBS); (**C**,**D**) undoped nanoparticles (NPs); (**E**,**F**) calcium NPs; (**G**,**H**) zinc NPs; (**I**,**J**) doxycycline NPs. A mature biofilm could be seen in the negative control group (**A**,**B**). In unloaded NPs, calcium NPs and zinc NPs (**C–H**), there were similar biofilm structures and development, with isolated areas of the discs free from biofilm growth. Doxycycline NPs was the only group without an observable biofilm on the coated titanium surface. Magnification: (**A**,**C**,**E**,**G**,**I**) 1000×; (**B**,**D**,**F**,**H**,**J**) 3000×.

At 72 h, a mature biofilm was present in the negative control group (Figure 4A,B). In addition, on the TiDs with Un-NPs, Ca-NPs, and Zn-NPs, the biofilms developed a similar structure, but still isolated areas of the TiDs were free from biofilm deposits (Figure 4C–H). On the TiDs with Dox-NPs, there was no biofilm formation (Figure 4I,J).

### 3.2. Analysis of Biofilms Vitality and Thickness by Confocal Laser Scanning Microscopy (CLSM)

**Figure 5:**
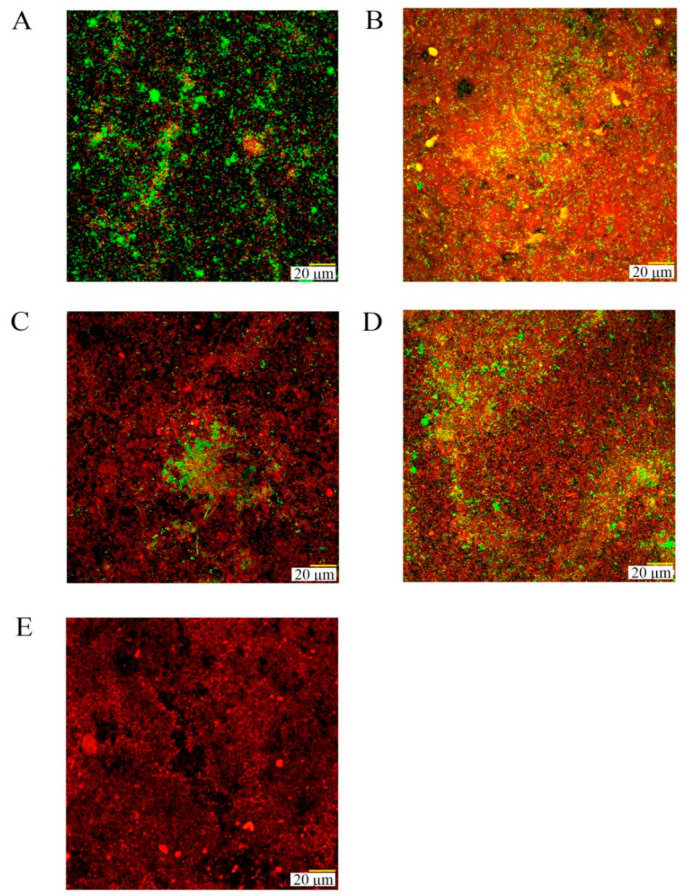
Confocal laser scanning micrographs after 72 h of growth on titanium discs (TiDs) coated with: (**A**) phosphate buffer saline (PBS) as control; (**B**) undoped nanoparticles (NPs); (**C**) NPs doped with calcium; (**D**) NPs doped with zinc; (**E**) NPs functionalized with doxycycline. A LIVE/DEAD^®^ BacLight^TM^ Bacterial Viability Kit was used to assess the vitality of cells. TiDs coated with NPs presented a reduced cell vitality in their biofilms as compared with control groups (living cells are presented in green and dead cells in red).

After 72 h of biofilm development, biofilms were similar in thickness in all groups (ranging between 16 and 23 μm) (*p* > 0.05), although with statistically significant higher dead cell biomass in the coated TiDs as compared with the negative control. Dead cells percentages were 70.9%, 80.7%, 70.5%, and 85.9% for Un-NPs, Ca-NPs, Zn-NPs, and Dox-NPs, respectively, versus 47.2% in the negative control (Figure 5). Biofilms in TiDs doped with Dox-NPs also developed a statistically significantly lower ratio of viable/dead cells as compared with the negative control group (Control 2.16 and Dox-NPs 0.21) (*p* < 0.01) (Table 1).

### 3.3. Bacterial Load and Presence of Specific Bacteria Analysis by DNA Isolation and Quantitative Polymerase Chain Reaction (qPCR)

The effect of the tested NPs on bacterial counts (CFUs/mL) of the six tested species grown in the biofilm model is shown in Table 2.

In 12-hour biofilms, the counts of specific bacterial species were significantly reduced in the Dox-NPs as compared with the negative control group for *F. nucleatum, A. naeslundii*, *S. oralis,* and *V. parvula (p* < 0.001). In the coated-NP groups, with Un-NPs, Ca-NPs, and Zn-NPs, there was a significant increase in the bacterial load of *S. oralis* (*p* < 0.001) as compared with the negative control group. No statistically significant differences were found among Un-NPs, Ca-NPs, and Zn-NPs.

In 24-hour biofilms, counts of specific bacterial species were significantly reduced in the Dox-NPs as compared with the negative control group, for all target species, except for *P. gingivalis* (*p =* 0.136). Dox-NPs reduced bacterial load in all cases (*p* < 0.001).

In 48-hour biofilms, all tested species, except *P. gingivalis* (*p* < 0.04), were significantly reduced with Dox-NPs as compared with the control group (*p* < 0.001).

In 72-hour biofilms, all tested species were significantly reduced with Dox-NPs as compared with the control group (*p* < 0.01).

## 4. Discussion

The present investigation, using an in vitro subgingival biofilm model, has demonstrated the antibacterial effect of coating TiDs with NPs, when NPs are loaded with doxycycline. Discs covered with Dox-NPs, as compared with negative control discs, demonstrated statistically significant reduced bacterial vitality, lower live/dead cells ratio, and significant reductions in bacterial load of all tested species, after 72 h. Furthermore, when observed with SEM, no relevant biofilm formation was identified on the Dox-NPs TiDs.

NPs have previously been found to be non-toxic and non-apoptotic after being tested with a human fibroblast cell line [19]. NPs are composed of 2-hydroxyethyl methacrylate, ethylene glycol dimethacrylate, and methacrylic acid, but the synthesis process is characterized by an efficient method, performed in the absence of harmful solvents or non-polymerized compounds, which later may interfere with cellular biological processes and cytocompatibility. Taking this into account, and assuming that dissolution of these particles is unlikely, and also considering that un-polymerized monomers are absent, all toxicity observed in bacterial cells may not be related to the polymer compounds of NPs, but to the number of ions or doxycycline loaded on NPs. It may also be that NPs are physically interrupting some biological bacterial processes.

In the Dox-NPs group, the 48- and 72-hour biofilms showed scarce biofilm formation, probably due to the presence of immature and weak biofilms, which become easily detached during SEM preparation, as previously reported in the literature [20]. In contrast, the morphology of the developed biofilms on the discs covered with Un-NPs, Ca-NPs or Zn-NPs were not significantly altered. Similarly, there were no differences in biofilm thicknesses as compared the different NPs, with and without doping, versus the negative control TiDs. This effect may be explained since the initial bacterial adhesion and growth is determined by physical forces, due to the tribological properties of the surfaces [26]. The tested titanium surfaces, covered or not by NPs, were made of grade 2 titanium Straumann SLA^®^ surfaces, with a mean roughness (Ra) of 1.50 μm ± 0.11 and a three-dimensional topography with vertical changes (Rz) of around 20 μm [27,28]. Roughness microtopography not only facilitates initial bacterial adhesion, but also serves as a surface protection for initial cell growth and biofilm development, thus, providing anchorage to the bacterial community [26].

Moreover, observations with CLSM also resulted in no significant differences in viable cells between the different NP-coated groups as compared with the negative control, although the percentage of dead cells and the dead/viable cell ratio were significantly higher in the Dox-NPs group. Sánchez et al. 2019 [20] found similar results, when these NPs were applied onto hydroxyapatite discs. However, the bacterial mortality in the present research was about 10% lower than in the previous report. This may be due to the higher roughness of SLA titanium disc as compared with hydroxyapatite discs [28]. Similarly, the Dox-NPs demonstrated, in the 72-h biofilms, a statistically significant reduction in bacterial load as compared with the control group. These results clearly demonstrate the antibacterial potential of these Dox-NPs, probably due to the bactericidal effect of the loaded antibiotic [29]. Doxycycline may act against most bacteria by inhibiting the microbial protein synthesis. The mechanism of action is a result of binding the ribosome, to prevent ribonucleic acid synthesis by avoiding addition of more amino acid to the polypeptide [29]. Interestingly, clinical trials have shown that the combination of local doxycycline application, as an adjunct with mechanical debridement, did not always show a significant effect in peri-implantitis management [30], which may be explained by a limited diffusion of the antibiotic in biofilms forming within titanium surface irregularities. This supports the idea that the slow release of drug delivered from the titanium surface, at the bottom of the biofilm, may be more appropriate for this treatment indication. The use of polyglycolic acid (PLGA) nanospheres loaded with doxycycline has been clinically tested in periodontitis and peri-implantitis patients with promising results [31,32]. Lecio et al. (2020) [31] used 20% doxycycline-loaded PLGA nanospheres, as an adjunctive therapy for periodontitis, and found positive results in terms of reductions in bleeding on probing, probing pocket depths, and clinical attachment loss. The NPs used in the present study are non-resorbable materials with a burst release of doxycycline up to 7 days, and a maintained release of about 8 μg/mL for 21 days [21]. This concentration is high above the minimum inhibitory concentration (MIC) reported by Kulik et al. (2019), as required to inhibit the growth of 90% of organisms (1 μg/mL of doxycycline) for *P. gingivalis* and *A. actinomycetemcomitans* in a planktonic state [33]. In a single species biofilm, *P. gingivalis* showed an increased MIC of 12.5 μg/mL for doxycycline, while the MIC for *A. actinomycetemcomitans* was 20 μg/mL [34].

Although Dox-NPs have shown antibacterial effects in previous reports [19,20], these antimicrobial surface delivery systems need to be constructed considering other characteristics, such as: (1) enhancing or, at least, maintaining the chemical and physical properties of the titanium surfaces; (2) non-cytotoxic effect to host tissues; and (3) predictable long-term drug release with an effective and stable drug concentration to avoid bacterial resistance [35,36]. Toledano-Osorio et al., in 2018 [21], showed that Zn-NPs in solution (10 mg/mL) produced a sustained release of Zn^2+^ for up to 28 days, reaching a peak of 0.044 μg/mL. In the case of Ca-NPs, the release was 2.03 μg/mL at 28 days. Interestingly, Navarro-Requena et al. (2018) showed that Ca^2+^ concentrations that ranged between 100 and 150 μg/mL exerted, in dermal fibroblasts, a higher metabolic state, migration, collagen production, and, in general, an increase in gene expression related to wound healing [37].

In the present investigation, NPs were doped with calcium, zinc, and doxycycline, and were tested for their anti-biofilm effect using a multispecies in vitro biofilm model. Most of the previously introduced treatments/coatings have been tested using mainly single-species biofilms [36], which may be useful for initial screening purposes, but proper evaluations should take into account the diversity of the oral microbiota [36]. Still, the results from the present investigation should be interpreted with caution due to the limitations of the in vitro model. In this case, only six bacterial species were used for biofilm formation. In addition, dental implants with different surfaces, with different roughness and metal alloys, are available in the market and the present results may only be valid for the specific titanium surface tested. This demonstrated antibacterial and antibiofilm effect of the NPs, especially those doped with doxycycline, should be further investigated in more advanced preclinical and clinical research models.

One of the main advantages of the tested NPs is that they may be easily employed clinically, for example, NPs suspended on PBS may be spread with a micro-brush onto titanium surfaces during a surgical intervention for the treatment of peri-implantitis. During surgery, once access is gained to the affected area, and once the affected/infected soft tissue is eliminated and the implant surface is disinfected/decontaminated, NPs may be easily applied onto the titanium surface.

Doped nanoparticles may have antimicrobial properties as well as anti-inflammatory and healing-promoting activities. In fact, zinc- and calcium-doped NPs may have the ability to sequester calcium and phosphate onto their surfaces, when immersed in simulated body fluid solution [17,18,19] and, hence, could promote bone regeneration. Similarly, doxycycline has a broad-spectrum antibiotic effect, as well as the ability to reduce bone loss [38] and promote bone formation by reducing inflammation and osteoclastogenesis [39]. It has been recently found that doxycycline may increase up to 20 times the gene expression of OPG/RANKL ratio in cultured osteoblasts, favoring bone formation [40]. It may also act as an immunomodulatory agent [41,42], promoting bone healing. These properties should also be investigated using the appropriate research models, since peri-implantitis is a chronic inflammatory disease.

## 5. Conclusions

Within the limitations of the present in vitro study, non-resorbable polymeric nanoparticles doped with doxycycline were able to decrease the bacterial load in biofilms and alter their dynamics of formation. These doxycycline functionalized nanoparticles should be further investigated as a potential useful tool in the treatment of peri-implant diseases.

## Figures and Tables

**Table 1:** Mean (standard deviation) of the viable and dead cell biomass, viable/dead cell ratios, percentages of dead and living cells, and biofilm thickness measured by confocal laser scanning microscopy (*n* = 6), for the different experimental groups. Titanium discs were treated with phosphate buffer saline (control) or covered with the four types of nanoparticles (NPs), i.e., undoped NPs (Un-NPs), NPs doped with calcium (Ca-NPs), NPs doped with zinc (Zn-NPs), and NPs doped with doxycycline (Dox-NPs).

	Control	Un-NPs	Ca-NPs	Zn-NPs	Dox-NPs
Viable cell biomass	68,750.66 (56,340.99)	96,387.27 (68,263.95)	89,579.52 (151,360)	67,989.72 (56,604.86)	30,954.77 (36,063.15)
Dead cell biomass	48,959.41 (45,304.36)	217,722.52 * (112,422.09)	214,538.38 * (102,620.69)	162,149.91 (107,249.13)	184,033.22 * (74,629.22)
Viable/dead ratio	2.16 (3.03)	0.47 (0.32)	0.34 (0.45)	0.41 (0.21)	0.21 (0.27) *
% Dead cells	47.21%	70.89%	80.70%	70.50%	85.87%
% Viable cells	52.79%	29.11%	19.30%	29.50%	14.13%
Thickness (μm)	23 (5.1)	21 (2.5)	23 (11.2)	22 (6.1)	16 (4.5)

* Statistically significant differences as compared with a negative control (*p* < 0.01). Numbers in parentheses are standard deviation values.

**Table 2:** Number of bacteria (colony forming units (CFU)/biofilm), expressed as mean and standard deviations (SDs) of *S. oralis, A. naeslundii, V. parvula, F. nucleatum, A. actinomycetemcomitans,* and *P. gingivalis* grown as multispecies biofilm at the different times of incubation, measured by quantitative real-time polymerase chain reaction (qPCR) (*n* = 9 for each incubation time and material). Titanium discs (TiDs) were used as a negative control as compared with TiDs coated with non-doped nanoparticles (Un-NPs), or doped with calcium (Ca-NPs), zinc (Zn-NPs), and doxycycline (Dox-NPs).

Bacterial Species	Time of Incubation	Number of Bacteria (CFU/Biofilm, Expressed as Mean (SD))				
		Control	Un-NPs	Ca-NPs	Zn-NPs	Do × -NPs
*So*	12 h	4.36 × 10^7^	7.99 × 10^7^	8.80 × 10^7^	1.05 × 10^8^	2.03 × 10^4^
		(1.12 × 10^7^)	(1.89 × 10^7^) *	(1.94 × 10^7^) *	(3.28 × 10^7^) *	(1.30 × 10^4^) *
	24 h	4.92 × 10^7^	1.08 × 10^8^	1.09 × 10^8^	7.99 × 10^7^	7.75 × 10^5^
		(3.92 × 10^7^)	(6.78 × 10^7^)	(7.19 × 10^7^)	(4.58 × 10^7^)	(1.19 × 10^6^) *
	48 h	4.02 × 10^7^	4.45 × 10^7^	5.45 × 10^7^	5.19 × 10^7^	6.63 × 10^4^
		(2.17 × 10^7^)	(4.65 × 10^7^)	(2.34 × 10^7^)	(3.07 × 10^7^)	(6.83 × 10^4^) *
	72 h	7.42 × 10^7^	1.33 × 10^8^	9.88 × 10^7^	1.37 × 10^8^	1.97 × 10^5^
		(3.19 × 10^7^)	(5.75 × 10^7^)	(3.59 × 10^7^)	(5.35 × 10^7^) *	(2.76 × 10^5^) *
*An*	12 h	3.47 × 10^5^	5.11 × 10^5^	4.98 × 10^5^	4.68 × 10^5^	3.72 × 10^4^
		(2.28 × 10^5^)	(3.62 × 10^5^)	(2.35 × 10^5^)	(2.41 × 10^5^)	(2.17 × 10^4^) *
	24 h	9.68 × 10^6^	1.10 × 10^7^	1.30 × 10^7^	7.21 × 10^6^	4.64 × 10^4^
		(8.94 × 10^6^)	(1.06 × 10^7^)	(1.49 × 10^7^)	(4.50 × 10^6^)	(9.98 × 10^3^) *
	48 h	4.14 × 10^6^	3.90 × 10^6^	4.37 × 10^6^	4.34 × 10^6^	5.10 × 10^4^
		(2.47 × 10^6^)	(2.90 × 10^6^)	(3.14 × 10^6^)	(2.92 × 10^6^)	(2.41 × 10^4^) *
	72 h	7.72 × 10^6^	8.87 × 10^6^	7.74 × 10^6^	9.71 × 10^6^	5.06 × 10^4^
		(4.92 × 10^6^)	(5.90 × 10^6^)	(4.73 × 10^6^)	(3.82 × 10^6^)	(9.75 × 10^3^) *
*Vp*	12 h	3.98 × 10^6^	4.67 × 10^6^	5.79 × 10^6^	1.28 × 10^7^	9.72 × 10^3^
		(5.02 × 10^6^)	(5.14 × 10^6^)	(6.80 × 10^6^)	(2.09 × 10^7^)	(5.71 × 10^3^) *
	24 h	2.71 × 10^8^	2.93 × 10^8^	3.87 × 10^8^	2.87 × 10^8^	3.15 × 10^5^
		(2.49 × 10^8^)	(2.43 × 10^8^)	(3.30 × 10^8^)	(3.15 × 10^8^)	(4.41 × 10^5^) *
	48 h	3.39 × 10^8^	3.03 × 10^8^	3.68 × 10^8^	3.06 × 10^8^	8.82 × 10^5^
		(2.88 × 10^8^)	(4.47 × 10^8^)	(2.19 × 10^8^)	(2.59 × 10^8^)	(1.17 × 10^6^) *
	72 h	8.41 × 10^8^	9.66 × 10^8^	5.55 × 10^8^	9.60 × 10^8^	6.87 × 10^5^
		(6.59 × 10^8^)	8.82 × 10^8^)	(2.75 × 10^8^)	(6.83 × 10^8^)	(7.52 × 10^5^) *
*Fn*	12 h	2.56 × 10^6^	5.93 × 10^6^	4.35 × 10^6^	8.63 × 10^6^	4.95 × 10^5^
		(1.40 × 10^6^)	(4.98 × 10^6^)	(2.58 × 10^6^)	(7.15 × 10^6^)	(2.71 × 10^5^) *
	24 h	6.41 × 10^6^	1.34 × 10^7^	1.02 × 10^7^	1.23 × 10^7^	2.17 × 10^5^
		(3.73 × 10^6^)	(1.16 × 10^7^)	(3.18 × 10^6^)	(1.03 × 10^7^)	(1.34 × 10^5^) *
	48 h	2.20 × 10^7^	2.45 × 10^7^	2.77 × 10^7^	2.65 × 10^7^	2.81 × 10^5^
		(1.85 × 10^7^)	(3.03 × 10^7^)	(1.86 × 10^7^)	(2.33 × 10^7^)	(2.95 × 10^5^)*
	72 h	3.95 × 10^7^	4.30 × 10^7^	3.19 × 10^7^	5.20 × 10^7^	2.78 × 10^5^
		(3.08 × 10^7^)	(3.32 × 10^7^)	(7.63 × 10^6^)	(3.07 × 10^7^)	(1.23 × 10^5^) *
*Aa*	12 h	1.18 × 10^7^	2.39 × 10^7^	1.93 × 10^7^	2.51 × 10^7^	9.16 × 10^5^
		(1.70 × 10^7^)	(3.41 × 10^7^)	(2.69 × 10^7^)	(3.75 × 10^7^)	(1.32 × 10^6^)
	24 h	2.48 × 10^6^	5.42 × 10^6^	5.04 × 10^6^	5.76 × 10^6^	1.25 × 10^5^
		(1.78 × 10^6^)	(3.61 × 10^6^)	(3.52 × 10^6^)	(4.63 × 10^6^)	(7.29 × 10^4^)*
	48 h	3.64 × 10^5^	5.88 × 10^5^	7.16 × 10^5^	5.60 × 10^5^	1.10 × 10^5^
		(2.46 × 10^5^)	(3.87 × 10^5^)	(6.27 × 10^5^)	(3.63 × 10^5^)	(6.76 × 10^4^) *
	72 h	8.70 × 10^6^	8.84 × 10^6^	3.97 × 10^6^	5.67 × 10^6^	9.82 × 10^4^
		(1.10 × 10^7^)	(1.70 × 10^7^)	(3.42 × 10^6^)	(4.60 × 10^6^)	(3.88 × 10^4^) *
*Pg*	12 h	5.60 × 10^5^	1.26 × 10^6^	1.17 × 10^6^	1.52 × 10^6^	3.30 × 10^5^
		(4.07 × 10^5^)	(4.74 × 10^5^) *	(6.64 × 10^5^)	(5.10 × 10^5^) *	(1.49 × 10^5^)
	24 h	8.22 × 10^5^	2.13 × 10^6^	2.21 × 10^6^	2.10 × 10^6^	2.47 × 10^5^
		(9.92 × 10^5^)	(1.84 × 10^6^)	(2.17 × 10^6^)	(2.05 × 10^6^)	(2.19 × 10^5^)
	48 h	1.81 × 10^6^	2.32 × 10^6^	2.65 × 10^6^	2.92 × 10^6^	2.71 × 10^5^
		(2.42 × 10^6^)	(3.18 × 10^6^)	(4.17 × 10^6^)	(5.33 × 10^6^)	(2.77 × 10^5^)
	72 h	2.18 × 10^7^	5.30 × 10^7^	5.11 × 10^7^	9.03 × 10^7^	5.75 × 10^5^
		(2.78 × 10^7^)	(7.75 × 10^7^)	(7.48 × 10^7^)	(1.34 × 10^8^)	(8.43 × 10^5^) *

* Statistically significant differences as compared with negative control titanium discs (*p* < 0.01). No statistical differences were found among Un-NPs, Ca-NPs, and Zn-NPs. Numbers in parentheses are standard deviation values. *So*, *Streptococcus oralis*; *An*, *Actinomyces naeslundii*; *Vp*, *Veillonela parvula*; *Fn*, *Fusobacterium nucleatum*; *Pg*, *Porphyromonas gingivalis*; *Aa*, *Aggregatibacter actinomycetemcomitans.*

## Data Availability

The data presented in this study are available on request from the corresponding author. The data are not publicly available due to general data protection restrictions.
